# Gleanings in Camp and Hospital

**Published:** 1874-03

**Authors:** F. K. Bailey

**Affiliations:** Knoxville, Tenn.


### GLEANINGS IN CAMP AND HOSPITAL.

BY F. K. BAILEY, M.D., KNOXVILLE, TENN., JANUARY, 1874.

CASE I.—GUN SHOT WOUND.—WOUND OF THE PERINEUM.—*June 26th,* 1861, *Friday.*—In camp, at Alton, Ill.; 20th Illinois Infantry. About 5 P. M., B. F. Titus, a member of Co. "D," while in his tent, was wounded by the accidental discharge of a musket in the hands of a comrade. The ball entered near the anus, upon the right side, and made its exit about an inch below the pubis, and the same distance from the mesial line. But little blood was lost, and the shock inconsiderable. Simple dressings were applied, and he rested through the night.

*June 27th,* A.M.—Complains but little, except from a blistered point upon the right ischiatic region, from the burning of the cartridge-paper.

The muzzle of the gun was not more than six or eight inches from his body when discharged.

*June 27th*, 9 P. M.— Passed urine at noon, and was comfortable all day. Gave sul. magnesia, and applied cool dressings.

*June 28th*, 9 A. M.— No constitutional disturbance; cuticle removed from the nates, which had been blistered; urinates easily; no alvine evacuation.

*June 29th*, 9 A. M.—No evacuation from rectum; pulse soft and natural; but little pain. Gave castor oil and spts. turpentine.

*July 1st*.—Bowels moved freely and easily, for the first time since the injury was received. From this time the wound healed kindly. The man, on his recovery, was detailed in the commissary department, and did but little laborious duty.

CASE II.—GUNSHOT WOUND OF FACIAL REGION.—In the same tent, and while in a stooping posture, either preparing or eating supper, very near the above-mentioned soldier, was R. I. Smith of the same Co. The ball, after traversing the person of Titus, struck Smith one inch anteriorly to the external meatus auditorius, on the right side, and about four or five lines below a line leading from the meatus to the external angle of the eye. The point of departure was about one-eighth of an inch above a corresponding point on the left side.

There was copious venous hæmorrhage from both orifices, but most from the point of entrance. Blood flowed freely into the posterior nares, and nearly strangled him, as he laid upon the back. A large quantity of blood was thrown from the mouth, both by vomiting and coughing.

As soon as he could swallow, two ounces of whisky and one-quarter grain sulph. morph. were administered After thirty minutes, blood ceased to flow, and he was comparatively comfortable, and in complete possession of his faculties. He was placed upon a cot, and cool applications directed.

*June 27th*, 8 A. M.—Rested after midnight very well; pulse 80, soft, but irregular; can converse clearly, but complains of intense soreness upon both sides of the face, but most on the right; tumefaction as low as the submaxillary region; very difficult to swallow or open the mouth; no hæmorrhage. 9 P. M.—Has lain very quiet during the day; swallows better than in the morning; has taken beef-soup and drink. Gave a dose of epsom salts.

*June 28th*, 9 A. M.—Rested last night; bowels open; breathing obstructed through the nostrils; pulse, 80, and soft; sleeps much of the time (from the morphine). 9 P. M.—Has rested well; right side of the face still swollen.

*June 29th* 1 P. M.—Face less swollen; suppuration from both orifices; bowels open; to have good diet, and quiet enjoined.

*July 1st*.—Able to walk about. On the 6th, the regiment left for St. Louis, and Smith, with Titus, both remained in camp.

Smith slowly recovered, so far as the healing process was concerned, but never was well while under my observation. He was discharged during the next winter, having remained with the regiment as a hospital attache. He went to Chicago, I think; but of his subsequent history nothing has come to my knowledge.

The above is from pencil-notes taken at the time, and still preserved. The cases were interesting to us, being the first casualties of the kind which occured in the noble Twentieth, which subsequently became so distinguished for valor in the field.

Surgeon Goodbrake was not near at the time of the occurrence, but came in a few minutes, as also did other medical officers of the brigade. There were serious fears entertained, for a few days, that both cases would result unfavorably; for they might very easily have done so.

In the case of Smith, the ball must have passed through the posterior nares without injuring the roof of the mouth. Although a severe wound, it could hardly have passed this at a more favorable place. An inch variation, in any direction, would have been very much worse.

CASE III.—GUNSHOT WOUND OF THE LIVER.—Asahel Douglass, Co. "C," 15th Illinois Infantry, was brought to Savannah April 7th, 1862, from the battle-field of Shiloh, and placed under my charge. He was very much depressed, pale, and evidently laboring under some shock.

On examination, found that a large ball had passed through the right side of the body, and the point of entrance, if I rightly remember, was anterior. Regionally considered, it was evident that the liver had been wounded, judging from the very copious discharge of bile, or, at least, a liquid of a golden yellow hue, mingled with blood.

The distressed young man was made as comfortable as possible under the circumstances. Stimulants and good food were given, and the openings dressed often. I had been in the habit of considering the biliary secretion as rather abundant, but, till observing this case, no idea had been formed as to the full amount.

Large compresses, placed upon both orifices, would soon be completely soaked; and our supply of material for dressings became so completely exhausted before the arrival of sanitary boats from the North, that I was obliged to use my own personal clothing in order to render the young man comfortable.

The case remained under my charge till the 15th, when, with many others, he was placed upon a boat and carried North. I learned that he died before reaching his home, in Rockford, Illinois. This was the only case of wound of the liver that I ever met with so soon after its occurrence. The general appearances accorded with those mentioned by Guthrie.

As in all penetrating wounds of the abdominal cavity, there was shock and depressed circulation. Death, in this case, probably resulted from the shock rather than the inflammation, although reaction might have come on in the space of eight or nine days.

In Circular No. 3, "Report of Surgical Cases in the Army," page 40, under "G. S. Wounds of the Abdominal and Thoracic Cavities," we find a record of eight cases where the liver was wounded. In these, one man lived twenty-three hours; one fifty hours; one died on the same day, and another on the day after. The others died immediately, or very soon after being wounded.

The most striking feature in this case was the great amount of bile which escaped. The quantity was, of course, only estimated by the cloths, which were soon saturated. The sufferer could not lie down without increasing the distress, and it was necessary to prop him up as well as our limited means would admit.

The amount of bile secreted in twenty-four hours has been variously estimated by physiologists. Twenty-four ounces is the amount stated by Haller; and, by Liebig, from seventeen to twenty-four ounces. Biddu and Schmidt estimate fifty-six ounces; and, so far as I could judge from the appearances, the latter amount is nearest to being correct.

CASE IV. — NON-PENETRATING WOUND OF THE CHEST.—BALL PASSING ALONG A RIB.—W. S. Vail, Co. "B," 20th Illinois Infantry, was brought to Savannah, from the Shiloh battle-field, April 7th. He was placed in a bunk on the floor, and examined as were the rest. There seemed to have been considerable shock, and subsequent depression. It was found that a ball had entered upon the right of the chest, about an inch and a half from the sternum, and three inches below the clavicle. The place of exit was at an opposite point on the back; and the first glance suggested a wound of the lung. But the characteristic signs of such an injury were wanting; and on tracing the hand from the point of entrance along the rib, a slightly elevated ridge was found, attended with soreness on pressure. It became certain that the missile had struck obliquely upon the chest, and passed completely around to the back.

The wounded places were dressed, and the man did very well for a week, when he became suddenly delirious, and well nigh unmanageable. He was tied hand and foot, to prevent his tearing away and running into the street, which he had done once or twice.

May 2d, he was placed upon the steamer Blackhawk, and taken North. I think he was carried to Quincy, and thence to the Insane Asylum at Jacksonville.

During the summer he returned to his Co., in good health, and was killed on 1st Sept., 1862, at the battle of Britton's Lane, West Tennessee. A notable fact connected with this case was the furious delirium.

The shock from the wound might have aroused some latent predisposition. Of his family history I could learn nothing. I do not think he was more than twenty years old; and his habits were not bad.

---

NATURALIZATION OF THE EUCALYPTUS.—At a late meeting of the Royal Botanic Society of London, it was stated that several specimens of the *Eucalyptus globulus* were growing in the new greenhouse of medicinal plants, in the Society's garden in the Regent's Park, one being fifteen feet in height. Experiments are being made to test the hardiness of the plant in the open air. In view of the wonderful properties claimed for this tree—counteracting or modifying malarial poison—its growth should be made the subject of experiment in many parts of this country besides California.—*New York Medical Journal.*

